# Multiple extracellular vesicle types in peritoneal dialysis effluent are prominent and contain known biomarkers

**DOI:** 10.1371/journal.pone.0178601

**Published:** 2017-06-08

**Authors:** Lachlan James Pearson, I-yanut Klaharn, Bussakorn Thongsawang, Wasin Manuprasert, Thunvarat Saejew, Poorichaya Somparn, Piyatida Chuengsaman, Talerngsak Kanjanabuch, Trairak Pisitkun

**Affiliations:** 1 Systems Biology Center, Research Affairs, Faculty of Medicine, Chulalongkorn University, Bangkok, Thailand; 2 Kidney and Metabolic Research Unit, Faculty of Medicine, Chulalongkorn University, Bangkok, Thailand; 3 Peritoneal Dialysis Excellence Centre, King Chulalongkorn Memorial Hospital, Bangkok, Thailand; 4 Bamphaeo Hospital (Prommitr Branch), Bangkok, Thailand; Hospital Universitario de la Princesa, SPAIN

## Abstract

Peritoneal dialysis inevitability results in activation of inflammatory processes and its efficiency is highly variable between patients. An improved method to isolate biomarkers and study pathophysiological mechanisms in peritoneal dialysis effluent (PDE) is expected to be of much benefit for the development of this treatment approach and help with patient management. Extracellular vesicles (EVs) are released as part of normal cellular processes. Their proteome is expected to reflect both type and health of their cell of origin. Although there is a significant interest in using EVs for “liquid biopsies”, little is reported of their presence or composition in plentiful dialysis waste fluids, including peritoneal dialysis effluent (PDE). Here we determined the presence of EVs in PDE and subsequently characterized their proteome. EVs were first isolated from PDE using differential centrifugation, then a further enrichment using size exclusion chromatography (SEC) was performed. The presence of EVs was demonstrated using transmission electron microscopy, and their particle counts were investigated using nanoparticle tracking analysis and dynamic light scattering. Using tandem mass spectrometry, marker proteins from three types of EVs i.e. apoptotic bodies, ectosomes, and exosomes were identified. The proteomic results demonstrated that the isolation of EVs by differential centrifugation helped enrich for over 2,000 proteins normally masked by abundant proteins in PDE such as albumin and SEC markedly further improved the isolation of low abundant proteins. Gene ontology analysis of all identified proteins showed the marked enrichment of exosome and membrane-associated proteins. Over 3,700 proteins were identified in total, including many proteins with known roles in peritoneal pathophysiology. This study demonstrated the prominence of EVs in PDE and their potential value as a source of biomarkers for peritoneal dialysis patients.

## Introduction

Currently, there are two major dialysis options for end-stage renal disease i.e. peritoneal dialysis (PD) and hemodialysis. Worldwide data indicated that the total number of patients receiving PD is markedly increasing [1]. Although the overall outcome is similar between the two dialysis approaches [2], PD has been reported to have a more favorable outcome compared with hemodialysis in the first few years after starting therapy. However, although results vary by studies, PD appears to subsequently lose its advantage over time [3]. The reason for the less favorable outcome over time could be associated with peritoneal membrane dysfunction leading to ultrafiltration (UF) failure. An exposure of the peritoneal membrane to bio-incompatible solutions during PD gradually results in changes in morphology and, eventually, failure of peritoneal membrane. Without healthy function of the membrane, the PD is inevitably ineffective. Furthermore, unresolved peritoneal inflammation and peritoneal accumulation of inflammatory cytokines have been demonstrated to contribute to propagated coagulopathy and serious encapsulating peritoneal sclerosis (EPS) in 0.7% to 7.3% of patients [4]. Due to a wide diversity of peritoneal function between patients, both before starting and in response to PD, detailed elucidation of these pathophysiological processes would provide a better understanding of UF failure, permitting therapeutic options to be refined.

The traditional method for evaluating the pathological damage of the peritoneum is an invasive peritoneal biopsy [5]. However, the peritoneal biopsy does not reflect the global changes in the peritoneum and is not feasible and safe to perform within the context of clinical practice [5]. Also, functional tests (peritoneal equilibrium test) cannot differentiate simple peritoneal sclerosis from EPS [6]. Study of extracellular vesicles (EVs) in PD effluent (PDE) may be an alternative approach, a so-called “liquid biopsy”, to investigate peritoneal membrane pathophysiology in lieu of performing a peritoneal biopsy. EVs are potentially attractive sources of biomarkers in complex body fluids and are increasingly being demonstrated to have significant roles in various physiological processes throughout the body [7]; however, none has yet demonstrated the presence of EVs in PDE. EVs are heterogeneous in nature often classified into apoptotic bodies, ectosomes, and exosomes according to their formation processes, sizes, and surface protein markers [8]. Exosomes are typically 50–100 nm in diameter, originating from multivesicular endosomes [9]. While ectosomes [8,9] and more recently described small apoptotic bodies are 160–500 nm in diameter [10,11] and originate from the plasma membrane blebbing. Each type of EVs has unique constituents and roles in various cellular processes. EVs hold great promise as novel biomarkers for clinical diagnosis and therapeutic opportunity [12]. However, it may be difficult to expand this technology in the field of PD since the concentration of EVs in the PDE is expected to be minimal because of a diluting effect of infilled PD fluid (8–10 L/day), thus an optimal procedure for isolating EVs is required. Here we demonstrate the combination of 2 methods, differential centrifugation and size exclusion chromatography (SEC), to retrieve the EVs from the PDE.

Proteomics-based analysis of PDE is a relatively new approach to studying the changes associated with PD [13]. Recent studies have shown altered proteomic profiles in various conditions of PD patients, including uremia [14], diabetes [15], peritonitis [16], abnormal peritoneal transport function [17], and with different peritoneal dialysis modality [18]. However, these studies extracted proteins in PDE by precipitation techniques resulting in high abundant diffusible proteins from blood circulation which interfered with the identification of low abundant, locally secreted, yet important, biomarkers [13]. Methods to overcome this contamination include introducing more sample purification or separation steps once the proteins are isolated using 2D gel electrophoresis separation, liquid chromatography, and then sample enrichment [13]. However, these methods result in protein loss, increased technical demand, and possibly less reproducibility. Recently, the microvesicle/exosome isolation has been used to analyze protein changes in other body fluids without additional protein purification.

In this study, we hypothesize that EVs are secreted from peritoneal resident cells and contain known biomarkers for PDE-related membrane injury. As a proof of concept, here we identified and characterized EVs from PDE of healthy PD patients using transmission electron microscopy (TEM), dynamic light scattering (DLS), and nanoparticle tracking analysis (NTA). Furthermore, we did proteomics and bioinformatics analyses to determine the number and type of proteins measurable within EVs isolated from the PDE using two methods of vesicle isolation.

## Materials and methods

### PD effluent (PDE) samples

This study was approved by IRB of the King Chulalongkorn Memorial Hospital (499/58). All participants provided their written informed consent to participate in this study. The ethics committees/IRBs approved this consent procedure. Overnight PDE was collected from 8 healthy PD patients (Table 1) who had both, no residual renal function and peritonitis within the past 1 months. A repeat of the analysis was done from 5 different patients at a later date. Dwell time was 8 hours for all patients. Low-density membrane fractions were isolated using the most common exosome isolation method as described [19]. Briefly, all samples (300 ml of PDE per sample) were centrifuged at 17,000 x g for 15 min at 4°C to remove whole cells, cell fragments, large debris, lamellar bodies, denser ectosomes, and macrovesicles (such as traditionally described apoptotic bodies; 1 to 5 μm in size [8]). The supernatants were then centrifuged at 110,000 x g_ave_ for 1 hour at 4°C to obtain low-density membrane pellets. The pellets were subsequently resuspended in 200 μl of PBS and stored at -20°C until further processing unless otherwise stated. Five patients used for repeat analysis had samples processed as above, and then 50% of the low-density pellet was solubilized in 400 μl of PBS and passed thru a size exclusion chromatography (SEC) column with 70 nm pore size as per company instructions for plasma (IZON Science Ltd). 500 μl fractions were collected. Fractions 1–5 (2.5 ml in total), 6–10 (2.5 ml in total), and 11–30 (10 ml in total) were combined respectively. Pooled fractions were concentrated with a 10-kDa filter (Amicon Ultra-4) by centrifugation at 4,000 x g for 25 minutes. Relative percentages of protein in SEC pooled fractions were 0.5% (fraction 1–5), 4.8% (fraction 6–10), and 94.7% (fraction 11–30). Total protein amount in the SEC vesicle fraction (fraction 6–10) was 26 ± 15 μg.

**Table 1:** Patient demographic data.

Group	Sex	Age (years)	BW (kg)	Hct (%)	DM		Duration of PD (Month)	Peritonitis-free interval (Day)
					Yes	No		
A	F	57	57	30.5		✓	84	61
A	M	44	52	30.3	✓		36	142
A	M	49	55	36.6	✓		12	No previous
A	F	69	53.5	31.4	✓		60	134
A	F	56	38.8	33	✓		7	34
A	F	64	75	33		✓	12	No previous
A	M	77	46.2	32	✓		6	No previous
A	M	55	61.8	26.7		✓	24	No previous
B	F	62	48.8	39.3		✓	60	930
B	F	65	40	39.3		✓	72	90
B	M	67	54	28.7	✓		18	No previous
B	F	40	51.6	25.6		✓	7	No previous
B	M	66	70.8	32.5	✓		8	No previous

***Abbreviations***: BW = body weight; Hct = hematocrit; and DM = diabetes mellitus

### Transmission electron microscopy

Negative staining electron microscopy was performed to identify and determine the size and morphology of vesicles from the low-density membrane pellets, and then again, with the pooled fractions from SEC. Each low-density membrane pellet was mixed with 100 μl of 4% paraformaldehyde. Then 10 μl of the mixture was spotted onto the parafilm sheet. A grid was later floated on the mixture drop for 10 min. Then the grid containing the vesicles was washed by floating on PBS and water droplets, respectively. The grid was stained by floating on 1% uranyl acetate droplet for 1 min and finally evaluated using TEM (Joel JEM-1400 Plus operated at 100 kV).

### Nanoparticle tracking analysis (NTA)

For size distribution analysis of the membrane-bound structures using a NanoSight NS300 (Malvern), vesicle samples were diluted 1,000 times in particle-free PBS. Videos of 25 seconds were recorded at camera level 12. Videos were analyzed at a detection threshold of 2 on the NanoSight Software 3.1 Build 3.1.54. Five samples were submitted for analysis; samples were read in duplicate.

### Dynamic light scattering (DLS)

DLS measurements were performed with a Zetasizer Version 7.04 (Malvern). Membrane/vesicle pellets suspended in PBS as described above were diluted 1:1000 in PBS plus 0.05% Tween-20. Machine settings were reflective index 1.334 and viscosity (cP) 0.8900, at room temperature. Seven individual low-density membrane samples were submitted for reading then additionally processed samples after pooling of SEC fractions (1–5, 6–10, and 11–30) from 4 patients were analyzed. Controls included a column flow thru before sample addition, PBS only, and samples with and without Tween-20 (0.05%). Each sample had three recordings; each recording was the average of ten measurements. An intensity above 0.5 background intensity was considered as a positive for size calculations of particle population.

### Western blotting

Western blotting was performed on SEC pooled fractions (1–5, 6–10, and 11–30) and low-density membrane pellets. Samples were mixed with 5X sample buffer (7.5% SDS, 10 mM Tris, pH 6.8) and heated for 5 minutes at 70°C. 20 μg of proteins was loaded per lane of a 10% polyacrylamide gel (except for the pooled fraction 1–5 in which less than 5 μg was loaded). SDS-PAGE was run at 130 volts until the dye reached the bottom of the gel. The proteins were transferred onto nitrocellulose membrane with trans-blot turbo (Bio-rad) at 1.0 A, 25V for 30 minutes. The membrane was blocked for one hour in TBST with 5% milk powder. 1:1000 mouse mAb anti-CD63 (Abcam ab193349) was added and incubated at 4°C overnight. After washing, HRP-conjugated secondary rabbit anti-mouse antibody at 1:5000 was incubated for 1 hour and bands visualized after washing with ECL Prime Western Blotting Detection Reagent (GE Healthcare).

### 1D SDS-PAGE and in-gel trypsin digestion

Each low-density membrane pellet was suspended in 1X Laemmli sample buffer (1.5% SDS, 10 mM Tris, pH 6.8). The proteins from 8 low-density membrane pellet samples were pooled equally, and 200 μg of the protein mixture was separated by 1D SDS-PAGE using a 10% polyacrylamide gel for 60 minutes at 100 volts. The gel was rinsed with ddH_2_O, stained with Imperial Protein Stain (Thermo Scientific) for 5 minutes at room temperature, then destained in ddH_2_O for 60 minutes at room temperature. Negative controls included PBS alone and PBS plus 0.05% Tween-20. For repeat analysis, the low-density membrane pellet or SEC vesicle fraction (fraction 6–10) samples from 5 patients were pooled equally, and 130 μg of proteins from each pooled sample was loaded onto a separate lane and run as above. In-gel trypsin digestion was performed as previously described [19]. In brief, the gel was cut into 40 fractions from top to bottom. Each gel fraction was destained, reduced with 10 mM dithiothreitol, and alkylated with 55 mM iodoacetamide. For trypsinization, 12.5 ng/μl trypsin solution was added to each sample and incubated overnight at 37°C. The supernatant was transferred to a new tube, and 50% acetonitrile/5% formic acid was added for peptide extraction. The peptides were desalted with C18 StageTips. The samples were dried and stored at -80°C for mass spectrometry analysis.

### Nano-liquid chromatography tandem mass spectrometry analysis and database searches

Peptides were separated by nano-liquid chromatography (EASY-nLC 1000, Thermo Fisher Scientific) coupled to a mass spectrometer (Q Exactive Plus Hybrid Quadrupole-Orbitrap, Thermo Fisher Scientific) through an EASY-Spray nanoelectrospray ion source (Thermo Fisher Scientific). The MS methods included a full MS scan at a resolution of 70,000 followed by 10 data-dependent MS2 scans at a resolution of 17,500. The full MS scan range of 200 to 2000 m/z was selected, and precursor ions with the charge states of +1 or greater than +8 were excluded. Normalized collision energy of HCD fragmentation was set at 28%. Raw LC-MS/MS files were searched by X! Tandem (CYCLONE, 2013.2.01) against human databases (ENSEMBL v.76 Homo sapiens GRCh38) plus common contaminants concatenated with their reversed sequences. A target-decoy approach was used to limit a false discovery rate (FDR) of the identified peptides to less than 1%. Parent and fragment monoisotopic mass errors were set at 10 ppm. Carbamidomethyl at cysteine was used as a fixed modification mass. Variable modifications were oxidation at methionine. A maximum of 1 missed cleavage sites was allowed. The mass spectrometry proteomics data have been deposited to the ProteomeXchange Consortium via the PRIDE partner repository (http://www.ebi.ac.uk/pride) with the dataset identifier PXD006371.

### Bioinformatics analysis

Manual assignment of apoptotic, ectosome, exosome markers, and highlighted proteins of interest was performed by cross-referencing with reviewed literature from NCBI protein database. The database from Qiagen (http://www.sabiosciences.com/rt_pcr_product/HTML/PAHS-090Z.html) was used for identifying referenced markers of epithelial to mesenchymal transition. Reactome Pathway Database (www.reactome.org/) was employed for categorizing proteins that are in the pathways related to peritoneal membrane injury and fibrosis e.g. TGF-beta pathway. Gene ontology as performed for cellular component analysis using David bioinformatics resource 6.8 [20]. Two protein lists generated from pooled low-density membrane pellet (background) and pooled SEC vesicle fraction (target) samples were compared using gene ontology enrichment analysis and visualization tool, GOrilla (http://cbl-gorilla.cs.technion.ac.il/) [21].

## Results

### Patient characteristics

PDE was taken from 8 stable PD patients to perform the isolation of EVs using differential centrifugation only (Group A), and additional samples from 5 patients with similar demographics were repeatedly analyzed with differential centrifugation followed by SEC (Group B). Table 1 shows demographic data for the patients. For Group A, the average duration of PD therapy was 30 ± 28 months. Systolic and diastolic blood pressures were 142 ± 18 and 82 ± 11 mmHg, respectively. For Group B, the average duration of PD therapy was 33 ± 30 months, and blood pressure was 135 (± 27)/ 73 (± 13) mmHg.

### Transmission electron microscopy

**Fig 1:**
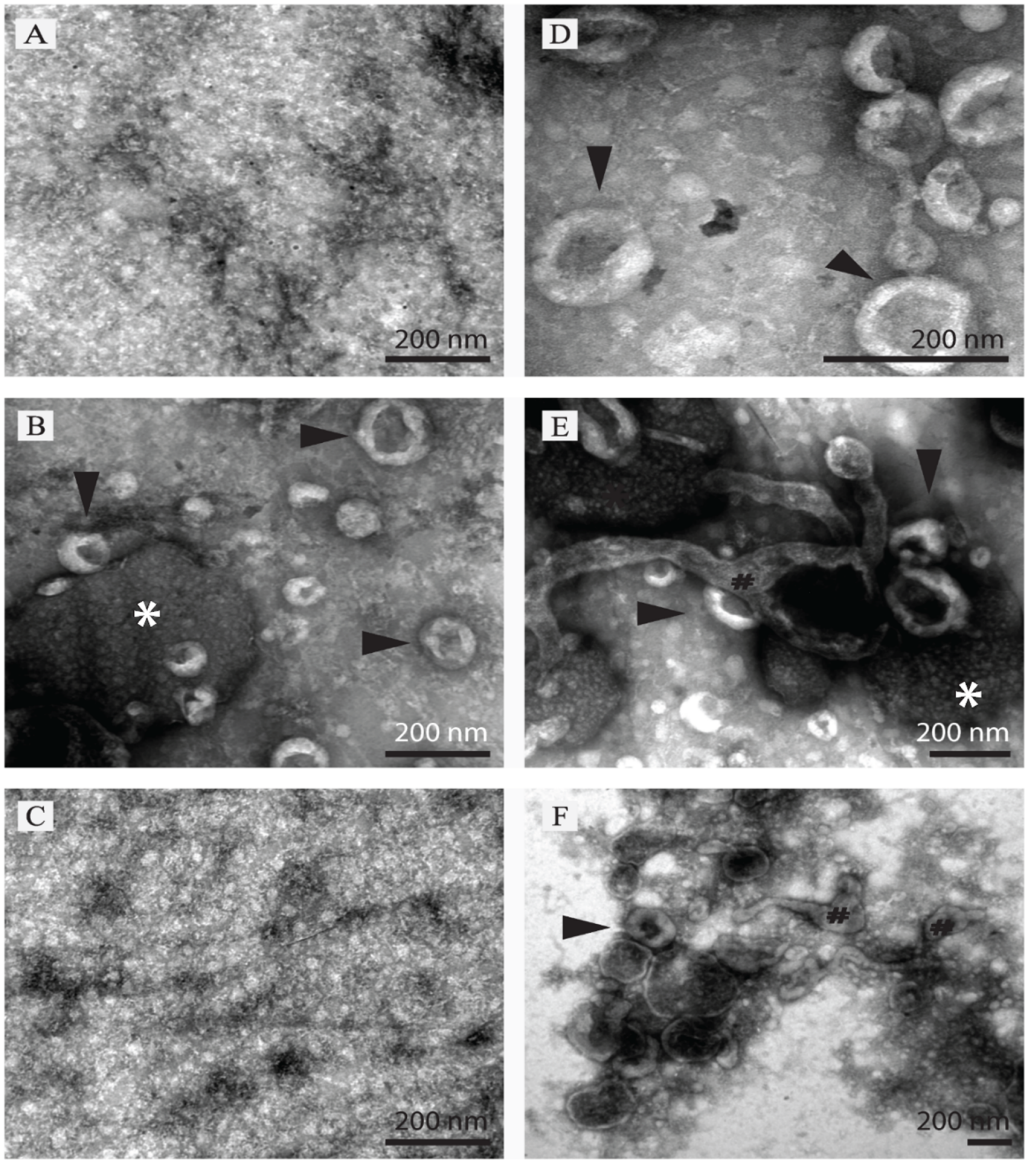
Transmission electron micrograph photos of 110,000 x g_ave_ pellet after size exclusion chromatography (SEC) (A-E) and without SEC (F). SEC pooled fraction 1–5 showed only grid background (A). SEC pooled fraction 6–10, vesicles (arrowhead) were in very high number and easily identified (B, D). Some other elongated membrane fragments (#) and unidentified large round particles (*) of approximately 250–500 nm were also observed in this fraction albeit very infrequent (E). SEC pooled fraction 11–30, small round particles less than 30 nm without visible membranes were observed under high background level (C). Low-density membrane pellet from non-SEC method had prominent background protein aggregates and other non-vesicle membrane particles mixed with vesicles (F).

TEM confirmed that there were vesicles, as defined by circular structures with a lipid bilayer without intracellular structures, from low-density membrane pellets or SEC fractions (Fig 1). After purification of the low-density membrane pellet by SEC, it was established that over 99% of eluted vesicles were present in the SEC pooled fraction 6–10 (SEC vesicle fraction) (Fig 1B). After SEC, it was also observable that there were two types of morphologically distinct particle, the majority being typical “exosome” in structure while there was also a significant population of unidentified large circular particles (Fig 1B). Size range after SEC was prominently between 50–200 nm typical for exosome type vesicles (Fig 1B and 1D). Note that the cup-shaped vesicles observed are a commonly reported TEM artifact of isolated exosomes [22]. The larger particles were 250–500 nm in size (Fig 1B and 1E). TEM of low-density membrane pellets without SEC purification (Fig 1F) indicated the majority of identifiable single structures over 30 nm in size were membrane-bound structures (50–600 nm); but many other background particles were also observed, sometimes partly obscuring vesicles, they may be protein aggregates, broken vesicles, or chylomicrons observable (Fig 1F).

### Particle count and size analysis

**Fig 2:**
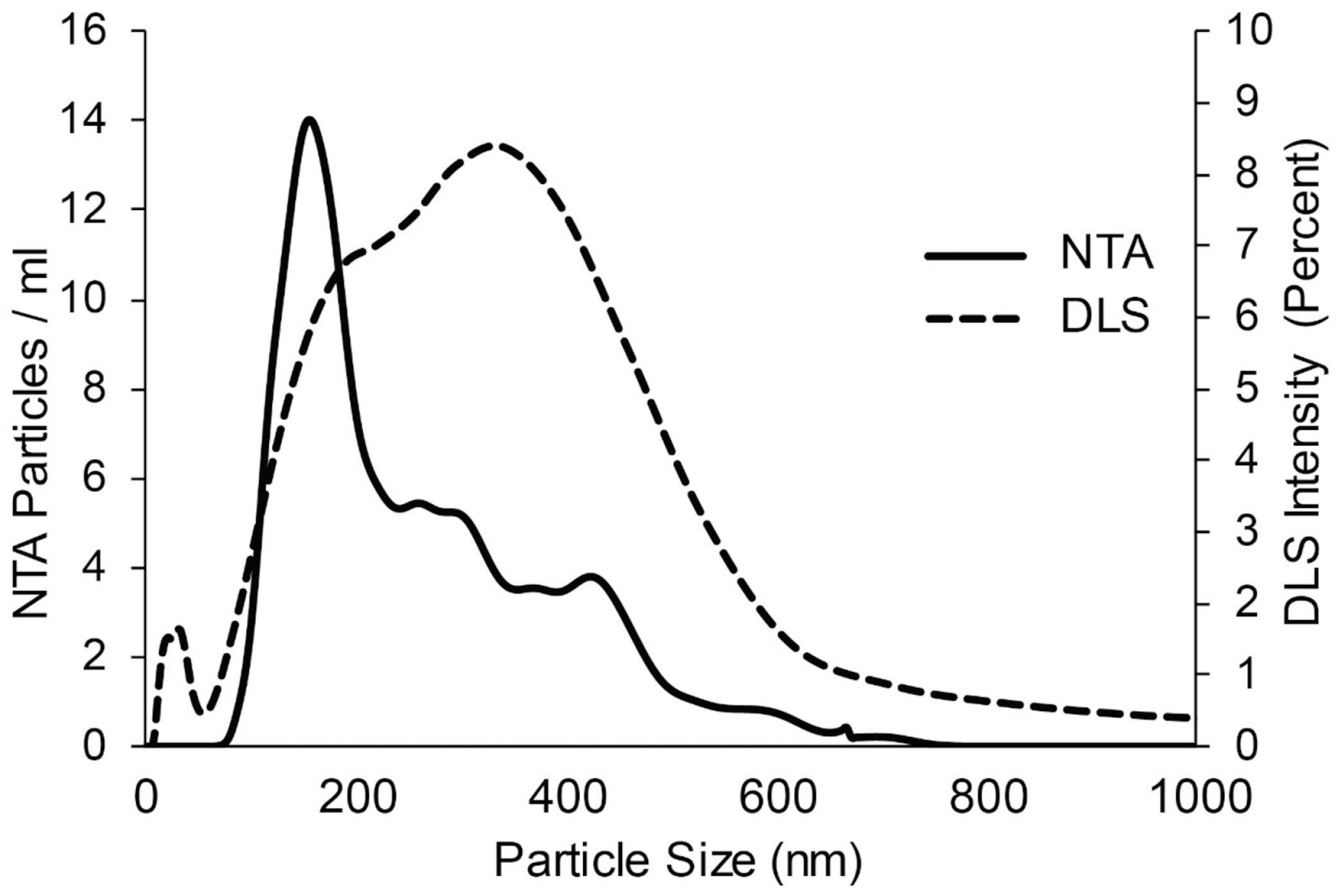
Particle size analysis. Particle size distribution was analyzed by nanoparticle tracking analysis (solid line) compared with dynamic light scattering analysis (dashed line).

Particle size distribution was evaluated by two methods NTA and DLS (Fig 2). NTA showed mean particle size of 213 nm (47–827 nm), mode of 155 nm, and 31% of particles less than 150 nm in size. Comparatively, DLS mean particle size was 282 nm (68–955 nm), and the mode was 342 nm. DLS percent intensity cannot be used to quantify vesicle numbers as larger particles give off more light. We found that NTA gave a mean particle size that was smaller and had narrower size distribution than DLS. A smaller population of 10–40 nm was seen with DLS. This population was also demonstrated in the negative control using a mixture of PBS and Tween-20, but not in PBS alone and therefore not included in calculations. Thus, this small population observed by DLS, but not NTA, was likely to be predominantly Tween-20 micelles rather than small vesicles. After SEC, two particle populations were evident, i.e. 99 ± 36 nm and 413 ± 150 nm (n = 4), in the SEC pooled fraction 6–10 using DLS. Particles were also observed in the SEC pooled fraction 11–30 but only when PBS was used without Tween-20.

### 1D SDS-PAGE and western blotting

**Fig 3:**
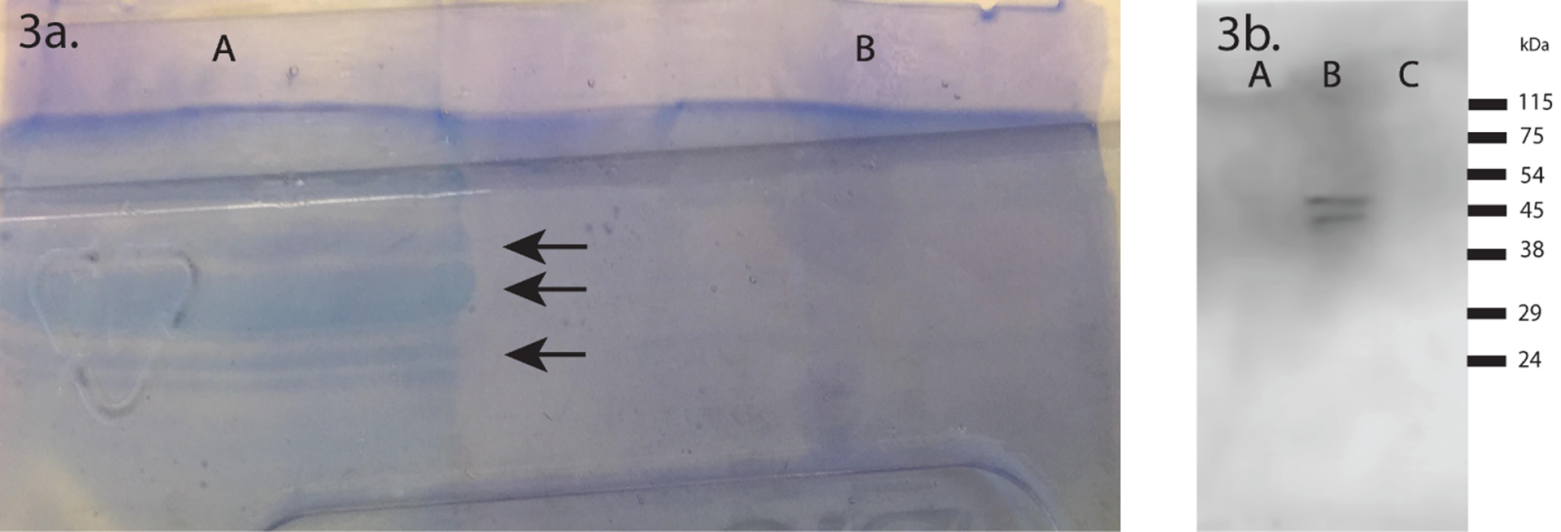
A) Coomassie stained gel used for mass spectrometry analysis. High MW bands were readily apparent (arrows) in the pooled low-density membrane pellet sample (lane A). After processing the low-density membrane pellet by SEC, high MW bands disappeared (lane B). B) Western blot for CD63. Two bands of approximately 50 kDa were seen with the SEC vesicle fraction (fraction 6–10) (lane B). The low-density membrane pellet (lane A) and SEC fraction 11–30 (lane C) samples showed no bands with the anti-CD63 antibody.

After Coomassie blue staining, several high MW bands were visible in the low-density membrane pellet lane (Fig 3A, lane A), but were absent in the SEC sample lane (Fig 3A, lane B). Western blotting showed that CD63 was detected in the SEC pooled fraction 6–10 sample, but not in the pooled low-density membrane pellet or SEC fraction 11–30 sample (Fig 3B).

### Proteomic profiling of extracellular vesicles

**Fig 4:**
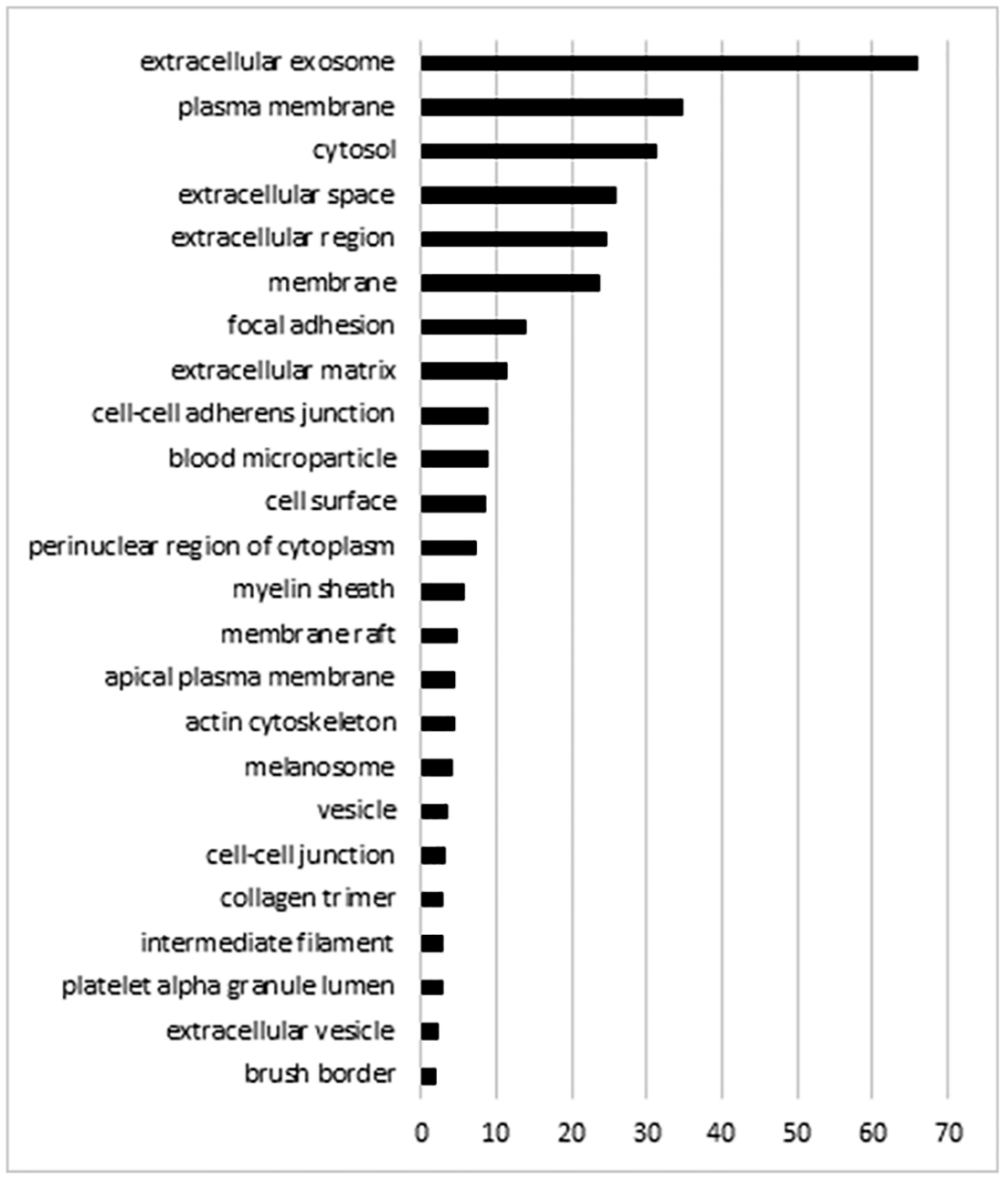
Gene ontology cell component analysis of proteins identified from low-density membrane pellet samples prepared by differential centrifugation. The analysis was performed using David bioinformatics resource. Proteins may be in more than one group.

**Fig 5:**
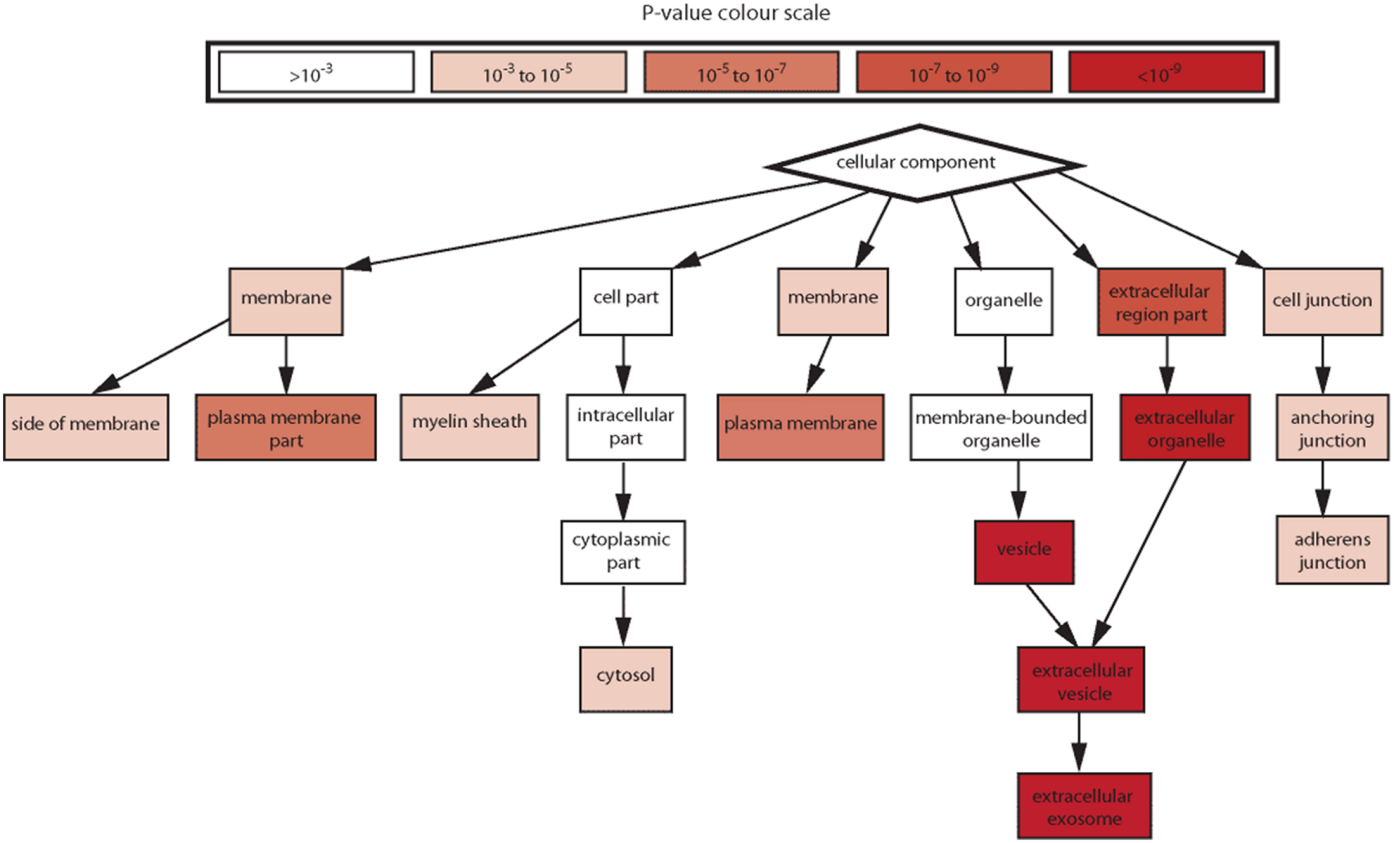
Gene ontology cell component analysis of proteins enriched by SEC. The protein list from the pooled low-density membrane pellet sample further purified by SEC compared with the protein list from the same sample without performing SEC showed a significant enrichment of membrane and vesicle terms, especially exosome. The analysis was performed using Gorilla.

A total of 3,744 proteins were identified by LC-MS/MS. All data can be accessible from the online database of this study (URL: http://sysbio.chula.ac.th/Database/PDEV/) and from S1 Table. Among all of the proteins identified, 2,094 were identified from low-density membrane pellet samples prepared by differential centrifugation. The additional purification of low-density membrane pellets by SEC further helped discover 1,650 more proteins. Protein markers associated with EVs and peritoneal pathophysiological responses identified in this study are further described in more details below. Gene ontology analysis for the cell component class showed a prominence of the exosome and membrane-associated proteins (Fig 4). SEC further enriched for the exosome component (Fig 5).

#### Identification of extracellular vesicle markers

Twenty-five protein markers of exosomes, 13 protein markers of apoptotic bodies, and 6 protein markers of ectosomes were identified (Table 2) [23,24]. Relative estimation of protein quantity was done by dividing the total peptide spectral counts per protein by its MW (normalized spectral count). Proteins were ranked based on the combined normalized spectral counts within the same class, see the classification in Table 2. After SEC, it was apparent that exosome markers were more prominently enriched, estimated at over 6 folds. In contrast, apoptotic body markers were less enriched and sometimes decreased. Protein enrichment ratio by SEC was determined by calculating the ratio of the normalized spectral count of SEC sample to the normalized spectral count of low-density membrane pellet (LDP) sample pooled from five patients (Table 2, Column 4). Exosome markers, TSG101 and CD63, were only identified in the SEC sample, but not in the LDP sample (see the online database).

**Table 2:** Apoptotic body, ectosome, and exosome vesicle markers identified in low-density membrane pellets.

Protein	Combined Spectral Count Frequency (Rank)	Normalized Spectral Count LDP*	Ratio Normalized Spectral Count SEC/LDP**	Gene Symbol	Vesicle Markers (Enriched)
C3b	14.40 (1)	14.4	1.4	C3	AB
H4a	11.83 (2)	4.74	1.2	H4a	AB
H2bf		2.37	2.5	HIST2H2BF	AB
H2ab		1.42	5.2	H2ab	AB
H2ac		1.36	2.4	HIST2H2AC	AB
H3		1.1	1.5	H3pseudo 2	AB
H3		0.32	3.2	HIST3H3	AB
H1c		0.23	0.4	HIST1H1C	AB
H1e		0.09	0.3	HIST1H1E	AB
H2a		0.07	0.6	H2AFZ	AB
H3.3a		0.07	1.3	H3F3A	AB
H2		0.05	3.4	H2AFY	AB
Annexin2	3.11 (3)	3.11	4.5	ANXA2	Exo
Basigin	0.77 (4)	0.45	3.7	BSG	Ecto
Basigin		0.32	3.0	SERPINF1	Ecto
Gi2alpha	0.62 (5)	0.62	7.6	GNAI2	Exo
MHC Class I	0.61 (6)	0.02	SEC only	HLA-A	Exo
MHC Class I		0.42	7.7	HLA-A	Exo
MHC Class I		0.1	3.25	HLA-A	Exo
MHC Class I		0.07	14.4	HLA-B	Exo
CD81	0.60 (7)	0.5	4.4	CD81	Exo
CD81		0.1	0.5	CD81	Exo
HSP70	0.51 (8)	0.23	5.2	HSPA1A	Exo
HSP70		0.2	5.1	HSPA8	Exo
HSP70		0.04	SEC only	HSPA2	Exo
HSP70		0.03	1.2	HSPA6	Exo
HSP70		0.01	19	HSPA5	Exo
MFG-E8	0.36 (9)	0.26	8.3	MFGE8	Exo
MFG-E8		0.11	1.4	MFGE8	Exo
CD9	0.31 (10)	0.31	2.6	CD9	Exo
β1 Integrin	0.18 (11)	0.18	4.1	ITGB1	Ecto
MMP2	0.17 (12)	0.17	1.3	MMP2	Ecto
TR	0.13 (13)	0.13	9.8	TFRC	Exo
ARF6	0.10 (14)	0.1	2.9	ARF6	Ecto
ICAM-1	0.09 (15)	0.09	3.8	ICAM1	Exo
MHC-II	0.07 (16)	0.07	16.3	HLA-DRA	Exo
TSP	0.06 (17)	0.06	0.9	THBS1	AB
VCAM-1	0.05 (18)	0.05	4.0	VCAM1	Exo
HSP90	0.05 (19)	0.05	7.5	HSP90AB1	Exo
Clathrin	0.04 (20)	0.04	7.0	CLTC	Exo
Rab27a	0.04 (21)	0.04	SEC only	RAB27A	Exo
Alix	0.01 (22)	0.01	16	PDCD6IP	Exo
MUC1	0.01 (23)	0.01	1.1	MUC16	Ecto
ADAM	0.01 (24)	0.01	9.3	ADAM10	Exo

***Remarks***: Proteins markers were ranked according to protein class frequency. Normalized spectral count equals total spectral number divided by protein molecular weight. Ratio normalized spectral count SEC/LDP > 1 indicates that the protein is enriched by SEC.

*sample combined from 8 patients

**repeat experiment, combined from 5 different patients

***Abbreviations***: AB = Apoptotic body [32–34], Exo = Exosome [23,24,26], Ecto = Ectosome [23,24,26], TR = transferrin receptor, TSP = thrombospondin, LDP = low-density membrane pellet, and SEC = LDP further purified by size exclusion chromatography

#### Highlighted marker proteins for investigation of peritoneal pathophysiological responses

Table 3 shows previously reported proteins involved in peritoneal inflammation or fibrosis (TGF-β pathway, other cytokines, and chemokines), proteins altered in a transition from an epithelial to a mesenchymal state, other proteins reported as regulated during peritoneal pathophysiological processes, and proteins involved in coagulation pathway.

**Table 3:** Proteins related to peritoneal membrane injury, fibrosis, and ultrafiltration found in low-density membrane pellet samples.

Description	Gene Name	Regulation and/or Response
**Epithelial to Mesenchymal Transition**		
AHNAK nucleoprotein	AHNAK	Upregulated
Bone morphogenetic protein 1	BMP1	Upregulated
E-cadherin (epithelial)	CDH1	Downregulated
Collagen, type I, alpha 2	COL1A2	Upregulated, TGF response
Collagen, type III, alpha 1	COL3A1	Upregulated, TGF response
Collagen, type V, alpha 2	COL5A2	Upregulated
Guanine nucleotide binding protein	GNG11	Upregulated
Caldesmon 1	CALD1	Downregulated
Catenin	CTNNB1	Differentiation and Development
Desmoplakin	DSP	Downregulated with transition to myofibroblasts
Epidermal growth factor receptor	EGFR	Cell Growth and Proliferation
Integrin-linked kinase	ILK	Cell Growth and Proliferation
Platelet-derived growth factor receptor, beta polypeptide	PDGFRB	Cell Growth and Proliferation
Keratin 14	KRT14	Differentiation and Development
Integrin, alpha V	ITGAV	Extracellular Matrix and Cell Adhesion
Integrin, beta 1	ITGB1	Extracellular Matrix and Cell Adhesion
Fibronectin 1	FN1	Extracellular Matrix and Cell Adhesion
Matrix metallopeptidase 2	MMP2	Extracellular Matrix and Cell Adhesion
Matrix metallopeptidase 9	MMP9	Extracellular Matrix and Cell Adhesion
TIMP metallopeptidase inhibitor 1	TIMP1	Extracellular Matrix and Cell Adhesion
Keratin 19	KRT19	Estrogen Receptor Signalling Pathway
Keratin 7	KRT7	Cytoskeleton
Moesin	MSN	Migration and Motility
Ras-related C3 botulinum toxin substrate 1	RAC1	Morphogenesis
**Chemokines and Cytokines**		
Complement component 5	C5	Chemotaxis
Adiponectin, C1Q and collagen domain containing	ADIPOQ	Cytokine
Macrophage migration inhibitory factor	MIF	Cytokine
Glucose-6-phosphate isomerase	GPI	Growth Factor
**TGF Pathway**		
Bone morphogenetic protein 1	BMP1	TGF superfamily ligand
Collagen, type I, alpha 1	COL1A1	See above
Collagen, type I, alpha 2	COL1A2	See above
Endoglin	ENG	Adhesion, Extracellular Matrix
Transforming growth factor, beta-induced, 68kDa	TGFBI	TGF superfamily ligand
Thrombospondin 1	THBS1	TGF superfamily ligand inhibitor/cofactor
Fas (TNF receptor superfamily, member 6)	FAS	Marker of TGF pathway activation
**Other Known Markers of Peritoneal Dialysis Pathophysiology**		
Aquaporin-1	AQP1	Increased expression can improve ultrafiltration
CRP	CRP	Sensitive marker of peritonitis, upregulated with ultrafiltration failure
Heat Shock Protein	HSP70	Marker of Renal Failure
Acute Phase Protein	ITIH4	Down-regulated chronic peritonitis
**Mesothelial cell marker**		
Mesothelin	MSLN	Mesothelial cell marker
Cancer Cell Antigen 125	MUC16	Mesothelial cell marker—cell mass, turnover, death
E-Cadherin	CDH1	Epithelial cell marker, Downregulated with transition to myofibroblasts
Desmoplakin	DSP	See above
Cytokeratins	KRTs	Downregulated with transition to myofibroblasts
Claudin 15	CLDN15	Overexpressed diffuse peritoneal malignant mesothelioma
**Clotting Pathway Factors Summarized**		
Factor II,V, IX, X, XI, XII, XIII (A&B)	F2-F13	Coagulation pathway
Fibrinogen	FG(A,B,G)	Converted to fibrin
Plasma Prekallikrein	KLKB1	Blood coagulation
Kininogen 1	KNG1	Blood coagulation
Plasminogen Activator	PLAT	Fibrinolysis
Plasminogen	PLG	Fibrinolysis
Endothelial Protein C Receptor	PROCR	Anti-coagulant
Protein S	PROS1	Anti-coagulant
Antithrombin III	SERPINC1	Anti-coagulant
Heparin Co-factor II	SERPIND1	Anti-coagulant

## Discussion

Here we demonstrate the various type of vesicles isolated from a peritoneal dialysis effluent that had a unique subset of proteins with known interest for peritoneal pathophysiological changes. Although in general, dialysis fluids are plentiful and easily obtained from the patients, the vesicles have not hitherto been established in this biofluid. Previously, high amounts of microRNA were extracted from the peritoneal fluid using an exosome precipitation method [25,26] but vesicles themselves were not mentioned [27]. Perhaps the reason for underreporting of vesicles in the dialysis fluids is possibly due to an interference effect of excessive protein contaminants on the vesicle isolation, low abundant vesicles in the fluid, or vesicle membrane rupture during the process of protein isolation using membrane filtration technique [28].

To demonstrate the vesicles, the ultracentrifugation method was employed in the study. Instead of a single type of vesicle, a wide range of vesicles was isolated here, including exosomes, ectosomes, apoptotic bodies, and unidentified large round particles of approximately 250–500 nm. Adding size separation to the density isolation did not only recruit more vesicles but also help achieve a high purity of the vesicles.

Our methods to confirm and characterize the vesicles were in accordance with the standard techniques described previously [29], including using TEM combined with other techniques to determine vesicle size distribution such as NTA or DLS, and the identification of recognized protein markers using western blotting and mass spectrometry.

TEM confirmed that there were plentiful vesicles in the low-density membrane pellets as defined by circular structures with a lipid bilayer without intracellular structures. After SEC, over 99% of eluted vesicles were present in the fraction 6–10. The majority of vesicles were typical exosome in structure, ranging between 50–200 nm while there was also a significant population of unidentified large circular particles.

To quantify and characterize nanoparticles in these polydispersed samples, NTA and DLS were simultaneously performed. An advantage of NTA and DLS over TEM is that they allowed precise quantification of vast numbers of particles without fixation, which can cause shrinkage of the vesicle. Here we found that the 2 methods, NTA and DLS, were generally concordant in the determination of particle size distribution. NTA more accurately defines populations of vesicles in size range of 50–400 nm, while DLS detects particles as small as 5 nm and more accurately measures particles around 1,000 nm [30]. However, DLS has several limitations in analyzing a polydispersed sample as applied here i.e. large particles could interfere with size determination of smaller particles [30], a group of multiple vesicles could be evaluated as a single vesicle, and large protein aggregates could not be distinguished from the interested vesicles. With both techniques, approximately 20–30% of the particles could be characterized as exosomes based on a size of less than 150 nm. After the SEC, 2 populations of particles were detected with DLS, one that could be prominently exosomes and another one that contained larger particles, supporting the TEM finding. It is yet to be determined whether vesicle quantities in PDE have any clinical significance.

PDE is a relatively dilute biological sample compared with others such as plasma. Isolation of vesicles is a possible way to enrich for low abundance, albeit clinically relevant proteins in the PDE. The total number of proteins observed in PDE-derived vesicles by proteomics in the present study was vastly greater than that reported in other studies using total protein extraction [13,16,18,31]. A complete list of the proteins identified can be accessible from the online database (http://sysbio.chula.ac.th/Database/PDEV/). One explanation is that the vesicle isolation enriches unique subsets of low abundant proteins otherwise obscured by certain abundant extracellular proteins. In fact, the additional purification step by SEC nearly doubled the number of identified proteins, allowed several vesicle markers to become detectable, and eliminated most prominent protein bands of high MW in the Coomassie-stained gel. A comparison of gene ontology for cellular component groupings between proteomic profiling studies of peritoneal fluid using vesicle-enriched samples in our case and using samples prepared by total protein isolation in other reported studies [16,18,31] also shows several differences. In addition to a greater proportion of membrane proteins, we observed over one hundred vesicle proteins. Interestingly one study which isolated total proteins from PDE also found a high proportion of vesicle proteins (20% of 151 proteins) [18]. Together, these findings may support the idea that vesicle proteins are a prominent proportion of PDE proteins although this was not backed by all studies [16,31].

The most abundant vesicle markers as estimated by spectral counting from our mass spectrometry data were apoptotic markers. Specific protein markers for apoptotic bodies are currently the least well-characterized of all the vesicle types (a few reported ones are histones, C3b, and thrombospondin) [32–34]. Unlike other vesicle types, a significant proportion of apoptotic body markers were decreased after SEC. A possible explanation is several apoptotic body markers are part of protein aggregates that were removed by SEC. MMP-2, a unique protein for ectosomes but not exosomes [35], was observed in this study while unique exosome proteins (e.g. CD81, integrin 3A, and ADAM10) were also identified [35]. Commonly used markers to demonstrate exosome isolation i.e. CD9 and ALIX were also observed (although enriched but possibly not strictly unique to exosomes [33,35]). Other common exosome markers, TSG101 and CD63, were not found in the low-density membrane pellets unless vesicles were purified by SEC, indicating that they may be either masked by abundant proteins or low in abundance. The expression of both CD63 and TSG101 can be highly variable dependent on the cell type [26]. With SEC, non-circular/vesicle-like membrane structures were very infrequently observed, and vesicle markers were highly enriched in general, supporting that the protein markers identified are at least predominantly associated with vesicles. Of note, markers of HDL were also observed. Although smaller (8–10 nm), HDL is known to be mixed with exosomes on density gradients [36]. Other lipoprotein contamination is also likely; our data showed that background protein contaminates were prominent and difficult to be removed. Isolation of subsets of vesicles that display unique markers by immunoaffinity purification is an obvious possible future progression towards reliable diagnostic marker discovery in PDE.

Marker proteins observed can also indicate the cell types from which the vesicles were derived. Likely sources of vesicles in PDE include mesothelial cells, immune cells, endothelial cells, platelets, and the blood (via diffusion). Mesothelial cell loss is believed to be a common response to PD-related peritoneal membrane changes and is therefore of interest [37]. Two markers for mesothelial cells were observed, mesothelin and cancer cell antigen 125 (MUC16). vWF, a marker of endothelial cells, but also potentially released by platelets was also present and was enriched by SEC. Few specific markers of immune cells were observed, perhaps because we recruited the patients who were stable as supported by their low PDE leukocyte counts. An exception was MHC-II, which was estimated to be markedly enriched by SEC; it is usually expressed highly on antigen presenting cells, although endothelial cells can also potentially express it. CD109 was also observed; it is expressed on T-cells, but also platelets and endothelial cells. Other membrane-associated proteins not reported to be enriched in vesicles such as CD14 and its coactivator lipopolysaccharide binding protein were also identified and may indicate innate immune processes [38]. The two latter markers were enriched by SEC demonstrating their vesicle association.

Subsequently, we determined if the proteins isolated could be useful for investigating known pathophysiological changes to the peritoneum during PD. Dialysis fluids and uremic status cause inflammation and subsequent loss of mesothelial cell layer, submesothelial fibrosis, angiogenesis, and hyalinizing vasculopathy [39]. One possible initiating event in this process may be epithelial to mesenchymal transition (EMT) in which mesothelial cells are converted to myofibroblast-type cells [37,39]. Prominent markers of EMT observed in our study were epithelial-associated or markers of late EMT such as E-cadherin and extracellular matrix proteins collagen I and III, respectively. αSMA, a key marker of myofibroblasts, was highly enriched by SEC. The most critical component of this pathophysiology is believed to be TGFβ, its upregulation resulting in the most distinct form of submesothelial fibrosis and thickening [39]. In this study, we found at least 7 protein components of the TGFβ pathway. Clotting factor pathway proteins were also prominent in our study. Fibrin deposition may lead to encapsulating peritoneal sclerosis, especially when associated with peritonitis and therefore could be a marker of this process [4]. Many clotting factors are released into the blood from the liver as soluble proteins. It is, therefore, possible that they are contaminants within the vesicles derived from the blood circulation [34].

Finally, we searched for proteins isolated with known interest for clinical applications. In contrast to other studies, membrane proteins were most prominent, including aquaporin-1 not found with a conventional proteomic approach [18] and hence previously required a biopsy to measure. Aquaporin-1 may mediate 50% of ultrafiltration, is implicated in ultrafiltration failure and is important for angiogenesis and endothelial permeability [40]. However, there might be a limited utility of aquaporin-1 as a biomarker for ultrafiltration failure because aquaporin-1 is also highly expressed in RBCs and endothelial cells. Hence extracellular vesicles released from these cells can be co-isolated with those derived from the peritoneal membrane. Histone expression levels may be a useful marker as they are important inducers of immune response independent of infection including chemokine release. Histones are known to be involved with peritonitis and possible transition from mesothelial cells to myofibroblast type cells [41]. MUC16 raised much interest as a marker of mesothelial cell mass in PDE, but it may also be increased by other conditions such as cell death [42]. General markers of peritoneal pathophysiology were also observed such as CRP. CRP is the most commonly recognized marker of peritonitis which also has predictive value on mortality in patients with PD [43], although its usefulness due to lack of specificity has also been questioned [44]. PDE patient analysis for apolipoprotein A1 showed that it is expressed higher in peritoneal membranes with high transport rates [17] associated with loss of ultrafiltration and risk of mortality.

A limitation of this study is that a small number of subjects were recruited, and we do not characterize the variability of protein/vesicle expression between patients but rather tried to acquire a complete list of potentially isolated proteins in a patient population. Thus, a carefully selected and large group of patients would probably be required to confidently detect useful biomarkers. Finding suitable reference proteins may also be a challenge when working with PDE samples. Choice of vesicle isolation method affects vesicle quantity and purity, therefore it is difficult to standardize.

In summary, we did a novel characterization and demonstrated a mixed population of EVs in PDE. A relatively simple isolation, “liquid biopsy” for vesicles, in a non-invasive manner, enriches for many proteins with known potential for determining peritoneal health and dialysis success. We have therefore shown this may be a useful approach to study peritoneal membrane. A carefully characterized group of PD patients is required to determine if vesicle-associated proteins are a sensitive measure of peritoneal changes.

## Supporting information

S1 Table Peritoneal dialysate extracellular vesicle (200,000 x g) proteome database. (XLSX)
